# Influence of Cu doping on the local electronic and magnetic properties of ZnO nanostructures†

**DOI:** 10.1039/d0na00499e

**Published:** 2020-08-28

**Authors:** Richa Bhardwaj, Amardeep Bharti, Jitendra P. Singh, Keun H. Chae, Navdeep Goyal

**Affiliations:** Department of Physics, Panjab University Chandigarh 160-014 India rbhardwaj.phy@gmail.com +91 7589297611; Material Science Division, Inter-University Accelerator Center New Delhi 110-067 India abharti_phy@yahoo.com; Pohang Accelerator Laboratory, Pohang University of Science and Technology Pohang 37673 Republic of Korea; Advanced Analysis Center, Korea Institute of Science and Technology Seoul 02792 Republic of Korea

## Abstract

In this paper, we report the existence of defect induced intrinsic room-temperature ferromagnetism (RTFM) in Cu doped ZnO synthesized *via* a facile sol–gel route. The wurtzite crystal structure of ZnO remained intact up to certain Cu doping concentrations under the present synthesis environment as confirmed by the Rietveld refined X-ray diffraction pattern with the average crystallite size between 35 and 50 nm. Field emission scanning electron microscopy reveals the formation of bullet-like morphologies for pure and Cu doped ZnO. Diffuse reflectance UV-vis shows a decrease in the energy band gap of ZnO on Cu doping. Further, these ZnO samples exhibit strong visible photoluminescence in the region of 500–700 nm associated with defects/vacancies. Near-edge X-ray absorption fine-structure measurements at Zn, Cu L_3,2_- and O K-edges ruled out the existence of metallic Cu clusters in the synthesized samples (up to 2% doping concentration) supporting the XRD results and providing the evidence of oxygen vacancy mediated ferromagnetism in Cu : ZnO systems. The observed RTFM in Cu doped ZnO nanostructures can be explained by polaronic percolation of bound magnetic polarons formed by oxygen vacancies. Further, extended X-ray absorption fine-structure data at Zn and Cu K-edges provide the local electronic structure information around the absorbing (Zn) atom. The above findings for ZnO nanostructures unwind the cause of magnetism and constitute a significant lift towards realizing spin-related devices and optoelectronic applications.

## Abstract 2

Cu doping in ZnO modifies the electronic structure and the hybridization between Cu and O orbitals results in RTFM due to formation of BMPs.

## Graphical abstract

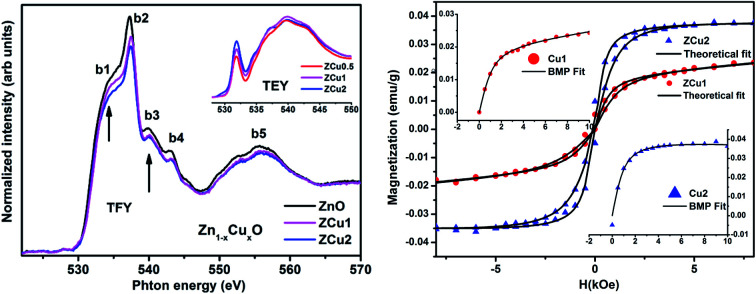

## 1 Introduction

Recent advancement in materials science has enabled researchers to synthesize novel semiconducting hybrid multifunctional materials which have unique physical, chemical, electronic, optical and magnetic properties. ZnO because of its wide bandgap, thermal and chemical stability, and large exciton binding energy outshines other oxide semiconductors for applications in diverse fields of electronics,^1^ spintronics,^2^ catalysis,^3^ sensing,^4^ solar cells,^5,6^*etc.* Nano-structuring also plays an important role in designing high performance devices with high sensitivity.^7^ ZnO has been reported for acquiring different nanostructures with different electrical and optical properties that are helpful in constructing photoelectric devices with an ultrahigh photo-response.^8^ On the other hand, the Al-doped ZnO (AZO)/NiO/AZO sandwich proves to be a heterostructure for soft transparent memristors.^9^ Besides this, for application in microelectronic and micro-electromechanical devices, lead free ceramics have been studied widely^10–12^ and it is reported that (Ba_0.98_Ca_0.02_)(Ti_0.94_Sn_0.06_)O_3_-*x* wt% ZnO ceramics where ZnO is used as a sintering aid have attained relatively high piezoelectric results.^13^

The conjunction of spin and charge states of an electron will be helpful in manufacturing low power consumption and fast processing devices. Transition metal (TM) doped ZnO is reported to have a high Curie temperature (*T*_C_) (above room temperature) both theoretically as well as from experimental illustrations.^14–16^ TM ions as dopants in the ZnO matrix modify its electronic and magnetic structural properties because the empty d states of TMs undergo hybridization with s or p states of the nearby anions influencing the electronic structure of the host lattice as it results in strong magnetic interaction between them.^17^ There are numerous reports in the literature on ZnO as a dilute magnetic semiconductor (DMS) stating its room temperature ferromagnetism (RTFM) property when doped with TMs and rare-earth metals.^18–22^ Ren *et al.* investigated the electronic and magnetic structure properties of 3d TM doped ZnO monolayers through first-principles calculations within density functional theory (DFT).^19^ A few reports also claimed the appearance of ferromagnetism in pure ZnO, termed as *d*^0^ magnetism.^23,24^ However, the issue that persists in the form of the nature of magnetism (whether intrinsic or extrinsic), its origin, and reproducibility is still under debate. TM ions in ZnO usually undergo phase segregation in the form of their oxides and sometimes form magnetic clusters/impurities which contribute to the magnetism while contradicting this, according to some experimental findings, the RTFM is purely intrinsic. A number of theoretical models have been proposed to unwind the origin of ferromagnetism in TM doped oxide semiconductors. First, Dietl *et al.* theoretically predicted the carrier (hole)-mediated exchange mechanism giving rise to RTFM in p-type wide band gap DMSs.^14^ But, this theory is contradicted by n-type DMSs having holes as minority carriers.^25,26^ Later Coey *et al.* proposed spin–split band theory in which shallow donors govern the magnetic moment in n-type wide band gap DMSs.^26^ Thus in DMSs, the type of carriers, their density of states and mobility tune the ferromagnetic properties. Besides this, it is also believed that the defects in the form of oxygen vacancies (*V*_O_), zinc vacancies (*V*_Zn_), oxygen interstitials (O_i_), zinc interstitials (Zn_i_), *etc.* affect the ferromagnetic ordering.^2,16,27,28^ The above issues are still fairly unresolved and thus detailed experimental and theoretical investigations are required especially to realize ion interactions inside the system *i.e.*, to probe the local environment around the host and the dopant atom carefully to obtain explicit results regarding the origin of FM in these systems.

Among various 3d transition metal ions, Cu doped ZnO has fascinating applications in optical switching as a magnetic semiconductor *etc.*,^29,30^ Cu^1+^ is diamagnetic and Cu^2+^ is paramagnetic in nature. A theoretical approach by Sato *et al.* suggested that the Cu : ZnO system is non-magnetic in nature with 25% Cu doping.^31^ Later studies approved that at low levels of Cu doping concentration ferromagnetism (FM) can be achieved.^32,33^ Hence, FM is highly sensitive to the synthesis route and nanoparticle growth environment and conditions. Liang *et al.* discussed thermally driven defect modulation in ZnO : Cu micron-scale polycrystalline films grown *via* spin-coating and observed dual-donor (Zn_i_ and *V*_O_) mediated ferromagnetic behavior in ZnO : Cu films.^34^ Besides this, Cu with rich optical properties has the advantage of tuning the bandgap and luminescence properties of ZnO. In the vicinity of Cu, the intensity of deep emission levels in ZnO increases.^35^ Thus, Cu doped ZnO as a DMS is supposed to have a longer coherence time which provides an opportunity for increasing the spin lifetime for practical spintronics applications.^36^ Furthermore, the literature states that the doped transition metal ion can change the magnetic properties of ZnO.^37,38^ However, the effect of doping is still complicated to understand. The surface effect and the growth of nanostructures under given synthesis conditions induce defects in the material and hence oxide based DMSs usually show different magnetic behavior.^39^ Thus based on the experimental results and theoretical calculations, this paper studies the influence of Cu doping on electronic structure and magnetic properties of ZnO.

In this paper, we used a facile and economic sol–gel method to synthesize Cu doped ZnO nanostructures at different Cu doping concentrations. The structural, optical, electronic and magnetic properties are studied in detail. Synchrotron radiation based X-ray absorption spectroscopy is a powerful technique to explore the defects/vacancies and local electronic structure in terms of their chemical states, oxidation number, and electronic transitions, which are supposed to be related to the origin of RTFM. The results obtained and their discussion unwind the cause of magnetism and prove ZnO to be a DMS having potential in spintronics and optoelectronic applications.

## 2 Experimental and characterization

### 2.1 Synthesis

Cu doped ZnO nanostructures were synthesized through a sol–gel route and analytical grade chemicals *i.e.*, Zn(NO_3_)_2_·6H_2_O (ZnNit), Cu(NO_3_)_2_·6H_2_O (CuNit), and NH_4_OH were purchased from Sigma Aldrich. Stoichiometric amounts of chemicals according to the formula Zn_1−*x*_Cu_*x*_O were taken at different concentrations of *x* = 0.005, 0.01, 0.02, 0.03 and 0.05. At first, appropriate amounts of ZnNit and CuNit were dissolved in de-ionized (DI) water corresponding to value *x*. To this solution aqueous NH_4_OH was added drop-wise (pH ∼ 9) and the precursor solution was continuously stirred on a magnetic stirrer with hot plate temperature around 70–75 °C. The resulting milky precipitates were centrifuged at 3000 rpm and washed several times with DI water and isopropyl alcohol. The sample obtained is oven dried at 80 °C for 6 hours and then annealed in a furnace at 400 °C for one hour at the step rate of 6 °C min^−1^. Finally it was ground into fine powder with a mortar and pestle. The samples are named as ZCu0.5 for *x* = 0.005, ZCu1 for *x* = 0.01, ZCu2 for *x* = 0.02, ZCu3 for *x* = 0.03 and ZCu5 for *x* = 0.05 and these names are used hereafter.

### 2.2 Characterization

An X-ray diffraction (XRD, Panalytical’s X’Pert Pro) spectrometer with Cu K_α_ radiation (*λ* = 0.1541 nm) was used to study the crystalline nature of the ZnO samples. A morphological study was carried out using a Field Emission-Scanning Electron Microscope (FE-SEM) (FEI Quanta FEG 200 HRSEM). For optical studies, a UV-diffuse reflectance spectrometer (UV-2600 SHIMADZU) is used and photoluminescence (PL) is measured using an FP-8500 spectrofluorometer. Magnetic data were measured through a Vibrating Sample Magnetometer (VSM) from the Department of Physics, Himachal Pradesh University, Shimla, India. Near edge X-ray absorption fine-structure (NEXAFS) measurements at O K- and metal (Cu,Zn) L_3,2_-edges were performed at the 10D (XAS-KIST) Pohang Accelerator Laboratory (PAL) beamline, South Korea and Taiwan Light Source (TLS), Taiwan. X-ray absorption near edge structure (XANES) and extended X-ray absorption fine structure (EXAFS) measurements for Cu and Zn K-edges were done at the 1D (XRS KIST-PAL) PAL beamline, South Korea.

### 2.3 Simulation details

FullProf software was used for Rietveld refinement of XRD data.^40^ FE-SEM micrographs were studied through ImageJ software. Simulation of XAS data was done using FEFF9.05 code based Athena and Artemis software.^41,42^

## 3 Results and discussion

### 3.1 Structure and phase analysis

**Fig. 1:**
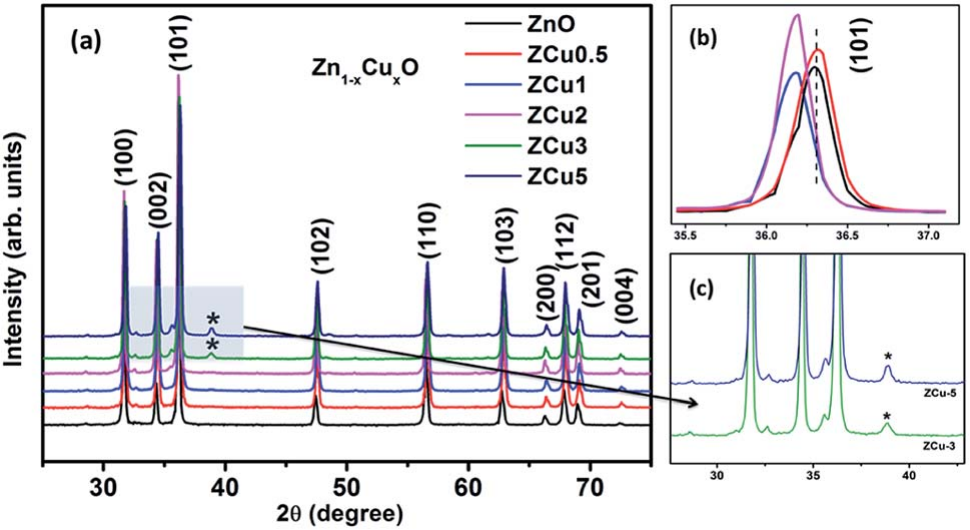
XRD patterns of Cu : ZnO systems at different Cu doping concentrations. The top right side shows the magnified view of the (101) plane. Below this is the magnified view of 3% and 5% Cu : ZnO systems in which * represents secondary phases other than the wurtzite structure of ZnO.

The characteristic XRD patterns of Cu : ZnO systems at different Cu concentrations are shown in Fig. 1. All synthesized samples exhibit highly intense peaks indexed according to JCPDS no. 01-089-1397 which corresponds to the formation of the hexagonal wurtzite structure of ZnO with the space group *P*6_3_*mc*. No additional diffraction peaks were observed up to *x* = 0.02 concentration emphasizing that the addition of Cu ions into the ZnO matrix did not affect the single phase of ZnO. However, for higher doping *i.e.*, *x* = 0.03 and 0.05, there is an extra phase appearing around 38° for Cu : ZnO systems on the 2*θ* axis and these small peaks are marked with asterisks (*). The reason for such peaks is the presence of nano-clusters of CuO(111) in ZCu3 and ZCu5,^43^ respectively. These doping concentrations (*x* = 0.03 and 0.05) are beyond the solubility limit for Cu in ZnO especially for synthesis through the sol–gel route. Thus, the remaining characterization experiments are carried out for the samples exhibiting the single phase wurtzite structure of ZnO. A visual examination of the XRD pattern further reveals that there is a peak shift towards left on the 2*θ* axis when compared with pure ZnO. The magnified spectra of the (101) plane are shown on the top right side of Fig. 1. This shift towards lower angles is due to the ionic radii difference of Cu^2+^ (0.071 nm) and Zn^2+^ (0.074 nm) ions. Doped Cu ions in the ZnO matrix substitute the Zn ion from its site and further, the peak shift indicates a change in lattice parameter values due to substitution.

**Fig. 2:**
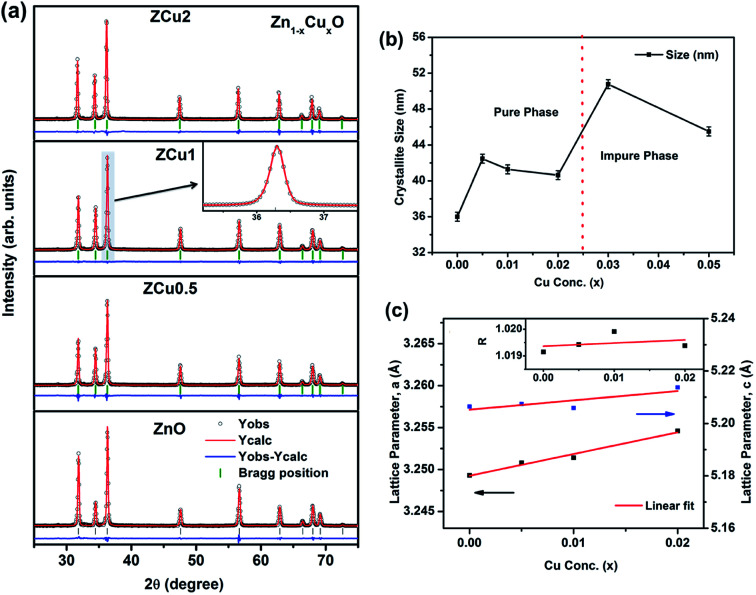
(a) Rietveld refined XRD pattern of pure and Cu doped ZnO samples; (b) variation in crystallite size with Cu concentration, *x*, estimated from the Scherrer equation; (c) variation in lattice parameters *a* and *c* on Cu doping. The inset of (c) represents the degree of distortion of the ZnO lattice on Cu doping.

To extract the detailed crystal structure, Rietveld refinement of XRD data has been performed for the single phase Cu : ZnO system. The steps for refinement are explained somewhere else.^44^Fig. 2(a) shows the Rietveld refined XRD patterns of Cu : ZnO systems at different Cu concentrations with the *χ*^2^ value less than 3 for all samples. Further, the crystallite size, *D*, is calculated using the Debye–Scherrer equation.^44^ The crystallite size for pure ZnO was found to be 36 ± 1 nm while on Cu doping the crystallite size was found to be in the range of 39–43 nm. Approximately the same crystallite size in the above cases is due to the minute difference in the ionic radii of Cu and Zn ions. The variation in crystallite size for Cu doping at concentration, *x*, is plotted in Fig. 2(b) and the lattice parameters, *a* and *c*, are plotted in Fig. 2(c). It is noticed from the lattice parameter plot that there is a small increase in *a* and *c* with increasing Cu-doping concentration in the ZnO matrix. The values of various reliability parameters and structural parameters are listed in Table S1.† The small linear variation in lattice parameters with increasing Cu ion concentration suggests that the doping does not affect the wurtzite structure of ZnO.^45^ The ideal wurtzite structure consists of hexagonal-close-packed (hcp) sub-lattices and has 
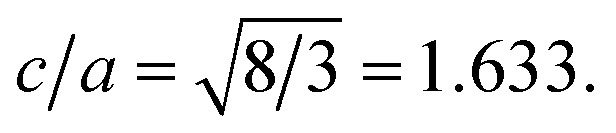
 The change in the cell parameter value can be attributed to the small lattice distortion of the Zn tetrahedron caused by the difference in the ionic radii of Cu ions present in the +2 oxidation state in tetrahedral coordination.^46,47^ The degree of lattice distortion for ZnO from its ideal tetrahedral coordination is calculated using the following equation1
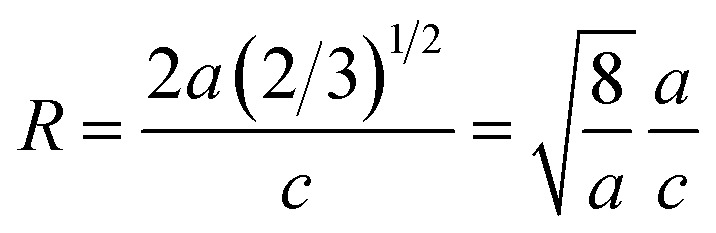
where *a*/*c* is the measure of distortion and *R* = 1 gives the ideal wurtzite structure.^48^ The graph for *R* is plotted in the inset of Fig. 2(c). The small linear increase of ‘*R*’ shows that the wurtzite structure remains intact on incorporation of dopant ions into the ZnO lattice. Thus, XRD and its Rietveld refinement confirm the formation of the single phase wurtzite structure up to a certain concentration of dopant ions and the formation of the ZnO nanostructure with a high degree of crystallinity.

### 3.2 Morphology study

**Fig. 3:**
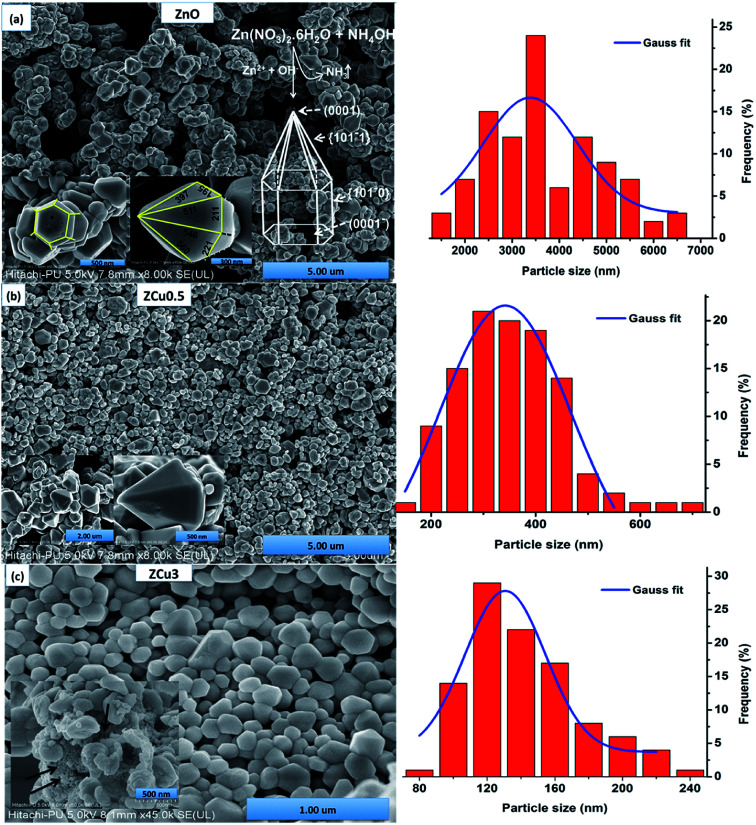
FESEM micrographs of (a) pure ZnO whose inset shows the magnified view of the hexagonal base and bullet-like structure labeled with sides whose dimensions are in nm along with the growth sketch of ZnO nanostructures; (b) ZCu0.5; (c) ZCu3. In the inset of (c) small-sized particles attached to the crystal surface (marked with an arrow) can be seen which represents the presence of impurities. The right side represents the particle size distribution histogram.

The morphologies of pure and Cu doped ZnO are analyzed through FE-SEM graphs, as shown in Fig. 3. For pure ZnO the well-defined faceted bullet-like nanostructure is formed under the controlled synthesis growth conditions *via* the sol–gel route. In Fig. 3, the particles circled with red color are the hexagonal base of the bullet-like structure and those circled with blue show the entire bullet-like shape. The inset of Fig. 3(a) shows the magnified view of the hexagon and hexagonal prismatic morphology. The sides of the tip are measured and marked with dimensions in nm. FESEM micrographs for the Cu : ZnO system with Cu concentration *x* = 0.005 in the ZnO matrix are analyzed and crystals quite similar to pure ZnO are observed, as shown in Fig. 3(b) and (c). The only difference is in the range distribution of size as can be seen from the histogram plotted. Moreover, the doped ZnO system appears to be much denser when compared to pure ZnO. With increasing Cu concentration to 3%, bullet crystals are deformed in shape and smaller size particles can be seen attached on the crystal surface, as shown in the inset of Fig. 3(c). These small-sized particles might be due to the presence of the CuO phase as observed in XRD results.

From the FE-SEM micrographs it is proposed that the growth of ZnO nanocrystals to form bullet-like structures must have gone through the following steps. At first Zn(OH)_2_ white precipitates were formed on titrating aqueous zinc nitrate with NH_4_OH (eqn (2)), which later on was transformed into a transparent solution of [Zn(OH)_4_]^2−^ on further heating.

2 Zn^2+^ + 2OH^−^ = Zn(OH)_2_

3 Zn(OH)_2_ = ZnO + H_2_O

It is a well known fact that the heat treatment of the material alters its physical and chemical properties. In our previous work, ZnO nanostructures were synthesized in an environment with hot plate temperature at 30 °C. Formation of small irregular shaped particles was observed.^44^ However, when the samples are annealed, as in the present case, these nanostructures change their morphology and grow into a bullet-like structure that depends on the growth rate of different ZnO crystal faces. Moreover, the increase in temperature results in a higher formation of nanoparticles because of a larger number of nuclei. With the heat treatment Zn(OH)_2_ dissociates to ZnO nuclei which are believed to be the primary units that lead to the growth of the final product. In general, ZnO is a polar crystal with the top plane being catalytically active Zn (0001) and the bottom being terminated by the O rich (0001̄) plane. Laudise *et al.* stated that the crystal growth is largely related to the difference in the rate of growth of various crystal facets in a given synthesis environment.^49^ The intrinsic anisotropy of the crystal lattice and the remarkable variations in surface energies between the planes {101̄0} and {0001} facilitate the crystal growth along the *c*-axis, which results in the bullet like ZnO nanostructure. To further confirm the elemental stoichiometry, the synthesized samples are investigated through energy-dispersive X-ray spectroscopy. Elemental analysis of ZCu0.5 confirmed the presence of Cu dopants and their composition in their respective ZnO systems, as shown in Fig. S1.†

### 3.3 Optical properties

**Fig. 4:**
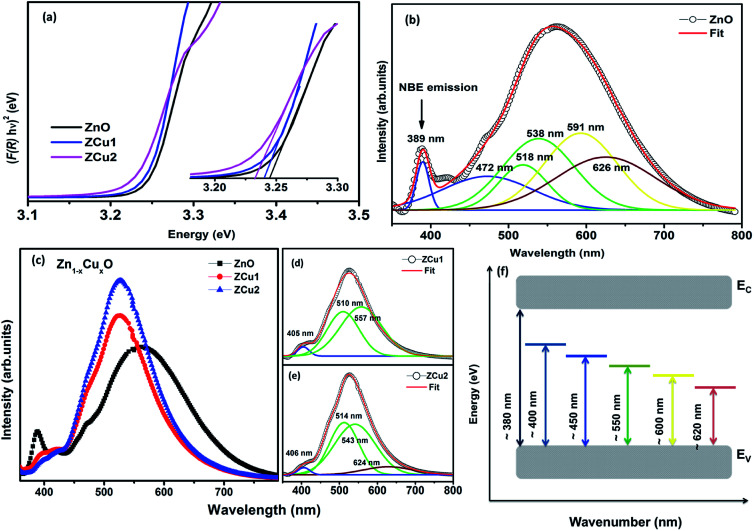
DRUV-vis spectra of (a) Cu doped ZnO nanostructures. The inset of (a) shows the magnified view of the same, (b) deconvoluted RT PL spectrum of the pure ZnO nanostructure, (c) RT PL spectrum of Zn_1−*x*_Cu_*x*_O nanostructures, (d) and (e) deconvoluted spectra for ZCu1 and ZCu2, and (f) energy band diagram based on PL data.

DRUV-vis measurements of the synthesized Cu doped ZnO powder samples are taken to study the optical properties. The Kubelka–Munk (K–M) relation (eqn (4)) is applied to determine the consequence of Cu doping on the energy band gap of the ZnO semiconductor.4
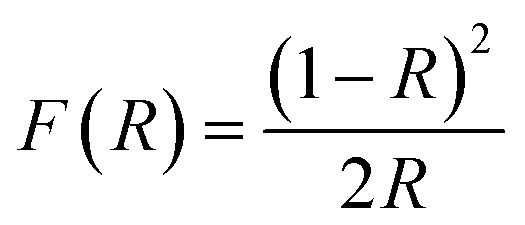
Here *F*(*R*) is the K–M function and *R* is the reflectance.^50^ For all samples, the graph is plotted between (*F*(*R*)*hν*)^*n*^ and *hν*, with *n* = 2 because of the probability of directly allowed transitions. The energy band gap value is obtained by linearly extrapolating the (*F*(*R*)*hν*)^2^ function to zero. A small decrease in the value of *E*_g_ was observed on Cu doping, as shown in Fig. 4(a). For Cu : ZnO systems, the bandgap decreases from 3.24 eV (ZnO) to 3.22 eV (ZCu2). Cu ions act as a donor impurity forming a shallow donor level just below the conduction band of ZnO and result in reduced bandgap energy. The curve shift indicates the successful incorporation of TM ions into the ZnO lattice and it is a result of exchange interactions between sp band electrons and localized d electrons of TM ions.^51^ Further, the PL technique is used to study the presence of structural defects and the degree of crystallinity of a material which highly influences the ferromagnetic properties in ZnO based DMSs. PL is one of the effective optical approaches to illustrate the presence of the intrinsic and extrinsic defects in a material. The PL measurements were carried out at room temperature for pure ZnO and Cu doped ZnO nanostructures at an excitation wavelength of 320 nm. The PL spectrum of the pure ZnO nanostructure exhibits a sharp peak around 389 nm (3.19 eV) and a broad emission peak in the visible region centered around 560 nm (2.21 eV), as shown in Fig. 4(b). The UV peak is attributed to the near band edge (NBE) emission which arises from the radiative recombination of free excitons.^52^ Also, the observed band gap for the ZnO nanostructure is found to be smaller than the band gap of bulk ZnO (3.37 eV) which indicates the presence of high defect levels in the synthesized ZnO material. The band gap narrowing is the result of donor impurities that create energy levels near the conduction band. The observed blue-green-yellow-red emission in the visible region is supposed to be due to deep level defect states/vacancies in the form of Zn_i_, *V*_O_, *V*_Zn_ and O_i_.^53^ Also, from the XRD results of the single phase of ZnO, it is interpreted that visible emission is caused by intrinsic defects only. Further, to illustrate which vacancy contributes to the visible emission in the region, the wide-range PL spectrum of ZnO from NBE emission to red emission is deconvoluted into six peaks centered at 380, 472, 518, 538, 591, and 626 nm using Gaussian fitting. The observed blue emission at 472 nm corresponding to 2.63 eV is attributed to the surface defects and electron transition from the donor level (Zn_i_) to shallow accepter levels (*V*_Zn_).^54,55^ The green emission centered at 518 and 538 nm is due to oxygen vacancies in the ZnO matrix.^52^ The yellow emission at 591 nm (2.10 eV) is associated with the recombination of the photo-generated hole and an electron trapped by 
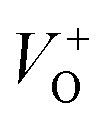
 vacancies in crystalline powder samples.^56^ In oxide semiconductors such as ZnO, oxygen vacancies are being considered as an important source of point defects and are generally present in three different charge states as neutral oxygen vacancies (*V*_O_), single charge oxygen vacancies 
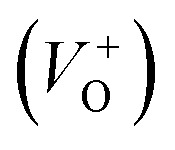
 and double charge oxygen vacancies 
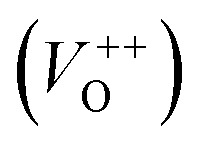
. The 
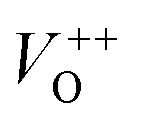
 and *V*_O_ vacancies do not contribute to the ferromagnetism in ZnO as they have spin-zero ground states.^57^ On the other hand, the 
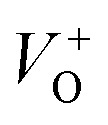
 vacancy plays a key role in activating the bound magnetic polarons (BMPs) and results in ferromagnetic ordering in DMSs.^58^ At last, the red emission at 626 nm (1.98 eV) corresponds to the presence of excess oxygen, O_i_ and Zn_i_ in the matrix.

RT PL spectra of Cu doped ZnO nanostructures are shown in Fig. 4(c). It is noticed that the Cu : ZnO system shows increased visible emission peak intensity which is considerably shifted towards lower wavelength with reduced peak width. The increase in PL intensity can be attributed to the increased number of defect states/vacancies. Just like the PL of ZnO, the peaks of the Cu doped ZnO system are deconvoluted using the Gaussian fit, as shown in Fig. 4(d) and (e). The UV NBE emission is observed at ∼405 nm for ZCu1 and ZCu2 which is shifted to the higher wavelength when compared to UV emission at 389 nm of pure ZnO. This verifies the Zn substitution by Cu ions in the ZnO matrix and is attributed to the Zn_i_ defects. On the other hand, the green emission observed for ZCu1 and ZCu2 is due to the formation of deep level emission (DLE) states between the valence band and conduction band. In the Cu doped ZnO case, the creation of DLE has been explained by Dingle *et al.* on the basis of the charge transfer phenomenon occurring due to the electron transition from the Cu^2+^ shallow donor state to the deep level oxygen atom acceptor state.^59^ This theory is further elongated by Graces *et al.* and it is suggested that if Cu exists in the +1 oxidation state, the emission follows the donor–acceptor pair recombination in which Cu acts as an acceptor and defects as the shallow donor state. However, if Cu is present in the +2 oxidation state it will give emission corresponding to Dingle.^60^ In the present case, NEXAFS results at the Cu L-edge show that Cu exhibits a +2 oxidation state and hence Cu plays a role of a shallow donor and accounts for the luminescence phenomenon which further intensifies the visible emission peak. Our results of visible peak shift towards shorter wavelength and increased intensity for the Cu : ZnO system are in accordance with the literature.^61^

### 3.4 Evaluation of vacancies and local electronic structure

The synthesized pure and Cu doped ZnO samples have been investigated through XAS which consists of both NEXAFS and EXAFS measurements. In NEXAFS, the transition of core electrons takes place into higher symmetry-allowed unoccupied bound states or low lying continuum states, which are unoccupied defect states and conduction band states in semiconductors. These transitions are governed through the dipole selection rule Δ*l* = ±1. NEXAFS spectra at O K-, and various metal L-edges *i.e.*, Cu L- and Zn L-edges have been recorded in total electron yield (TEY) and total fluorescence yield (TFY) mode. O K-edge NEXAFS spectral features, being very sensitive to the nearest neighbors, provide detailed and necessary points about the O (2p) state hybridization with different sp and d states of Zn and Cu doped ions.^62^

**Fig. 5:**
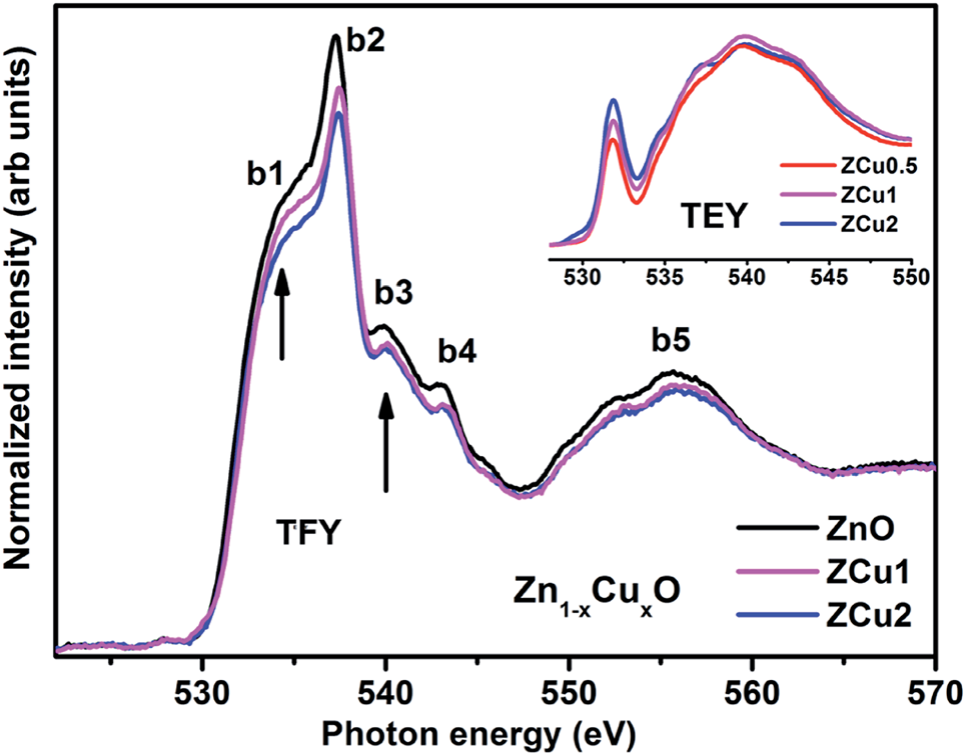
The experimental NEXAFS spectra recorded at the O K-edge for Cu doped ZnO nanostructures; the inset shows the data taken in TEY mode.

The normalized NEXAFS spectra at the O K-edge for Cu doped ZnO samples (ZCu0.5, ZCu1 and ZCu2) are analogous to that of pure ZnO with slight modifications in the spectral features, as shown in Fig. 5. The resemblance of the spectra is expected because the samples are probed at O atoms and not at other elements present in the system. However, the small variation in the spectral features is attributed to the change in electronic structure due to different Cu concentrations at the Zn position in different samples. Various spectral features observed in Cu : ZnO systems are marked as b1 at 533 ± 0.5, b2 at 537 ± 0.5, b3 at 539 ± 0.5, b4 at 543 ± 0.5 and b5 at 555 ± 0.5 eV, respectively. These features, b1–b5, are due to electronic transitions from the O (2s) orbital to Zn (4s4p)/Cu (3d4sp) orbitals. The spectral feature b1 is absent in pure ZnO and it becomes intense as the Cu doping concentration increases. The inset of Fig. 5 shows the O K-edge data measured in TEY mode where it can be clearly seen that the intensity of b1 increases monotonically with increasing Cu concentration. In pure ZnO, the Zn 3d orbital is fully filled and thus the transition from O (2p) to Zn (3d) is not feasible. On doping Cu into the ZnO matrix, the Cu ion substitutes the Zn from its tetrahedral site, and the empty Cu (3d) orbitals with *t*_2g_ symmetry become available for hybridization with oxygen orbitals. Higher the Cu ion concentration in the lattice, more will be the Cu (3d) empty states and intense will be peak b1. The pre-edge feature b1 corresponds to p–d mixing of O (2p) and Cu (3d) states, which is believed to be the reason behind the symmetry change from octahedral to tetrahedral and hence ferromagnetic nature of the Cu : ZnO system. The evolution of pre-edge spectral feature b1 delivers important information about the unoccupied states at the Cu (3d) level, which further reveals the presence of more charge carriers, electrons, or holes. Results similar to this have been reported in the literature for other oxide materials.^63^ The structural feature b2 is assigned to O (2p) state hybridization with Zn (4s4p) states. The spectral features b3 and b4 are assigned to O (2p) hybridization with Zn (4p)/Cu (4sp) states. Features b3 and b4 are related to oxygen-related defects in the ZnO matrix.^64^ Feature b5 in the broad region above 545 eV is almost similar and is independent of Cu concentration. Feature b5 arises mainly due to the O (2p) orbital hybridized with higher Zn orbitals and multiple scattering effects.

**Fig. 6:**
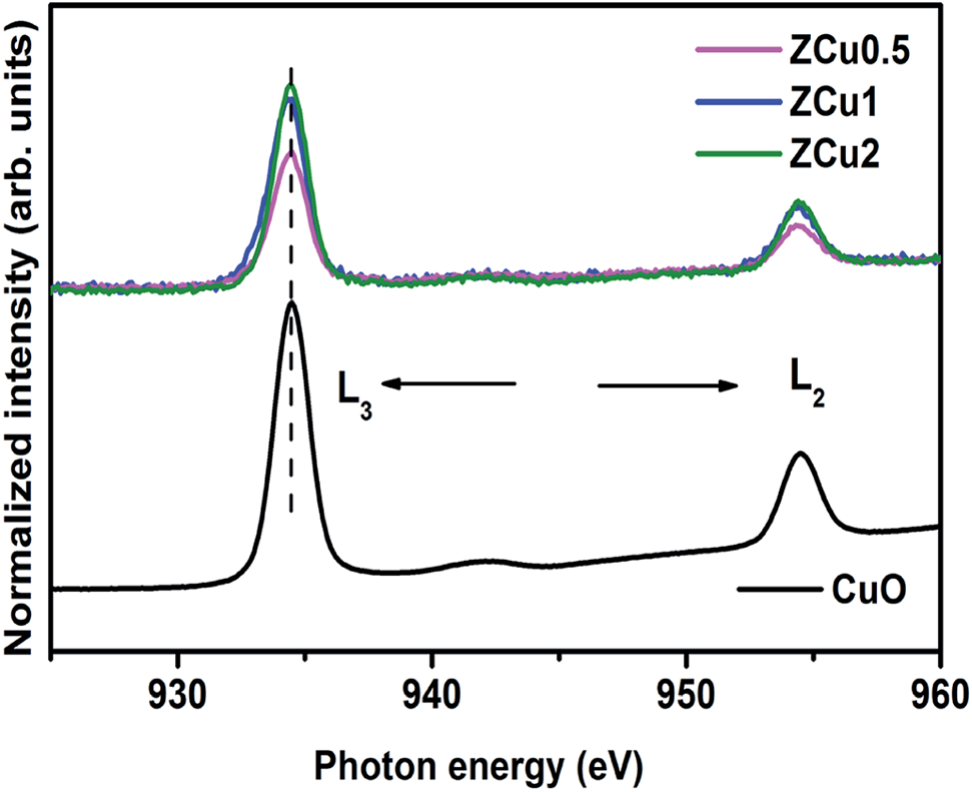
The normalized NEXAFS spectra measured at the Cu L-edge for Cu : ZnO systems. The data are compared with the L-edge of CuO taken as a reference sample.

Further, to elucidate the Cu oxidation state, NEXAFS spectra at the Cu L_3,2_-edge are measured for Cu-doped ZnO samples, as shown in Fig. 6. The spectral features in the region 932–938 eV marked as L_3_ are ascribed to the electronic transition from the 2p_3/2_ to the 3d orbital and those in the region 942–958 eV marked as L_2_ correspond to the 2p_1/2_ to 3d transition. These features emerge because of spin–orbit splitting of 2p core holes. These spectral features at the Cu L-edge of Cu doped ZnO samples are further compared with those of CuO taken as a reference sample. The resemblance of profile shape and line position with those of CuO confirms that Cu ions are present in the +2 oxidation state in the ZnO matrix.

**Fig. 7:**
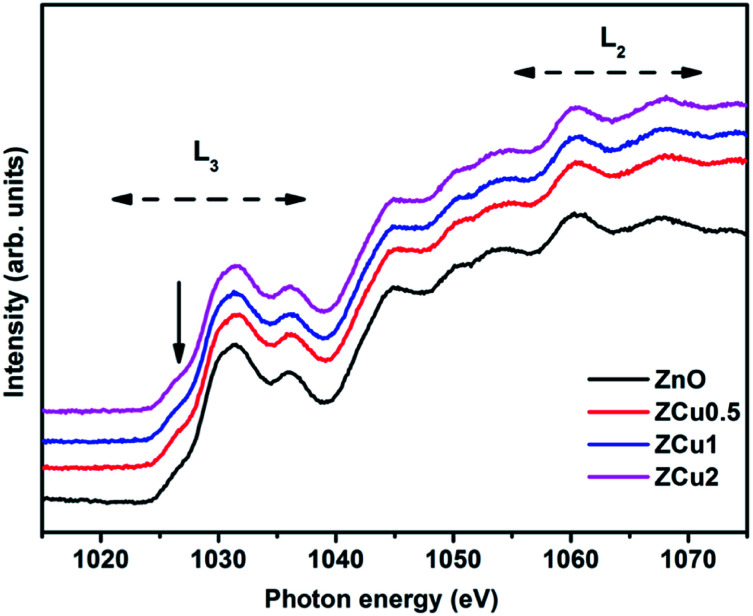
The normalized NEXAFS spectra at the Zn L-edge for Cu : ZnO systems.

The Zn L-edge is further probed through NEXAFS measurements, which provides information regarding the unoccupied Zn d and s states. Fig. 7 highlights the normalized Zn L_3,2_-edge spectra of Cu doped ZnO nanostructures. The two regions marked as L_3_ and L_2_ correspond to Zn (2p) to Zn (4s) and anti-bonding Zn (3d) states following the Mott-selection rules.^65^

The spectral features in the L_3_ region correspond to the electron transition from Zn (2p) to Zn (3d) states because 3d orbitals are more localized compared to 4s orbitals. However the pre-edge feature marked with the downward arrow is dominated by Zn 4s band transitions. The spectral features in the L_2_ region are due to multiple overlapping of bands.^66^ Further, it is observed that all the spectral features of pure ZnO and Cu doped ZnO are symmetrical in shape and position *i.e.*, there is no change in spectral feature intensity or evolution on Cu doping. This indicates that Zn related defects are not responsible for the observed room temperature magnetic properties of the Cu : ZnO system.

**Fig. 8:**
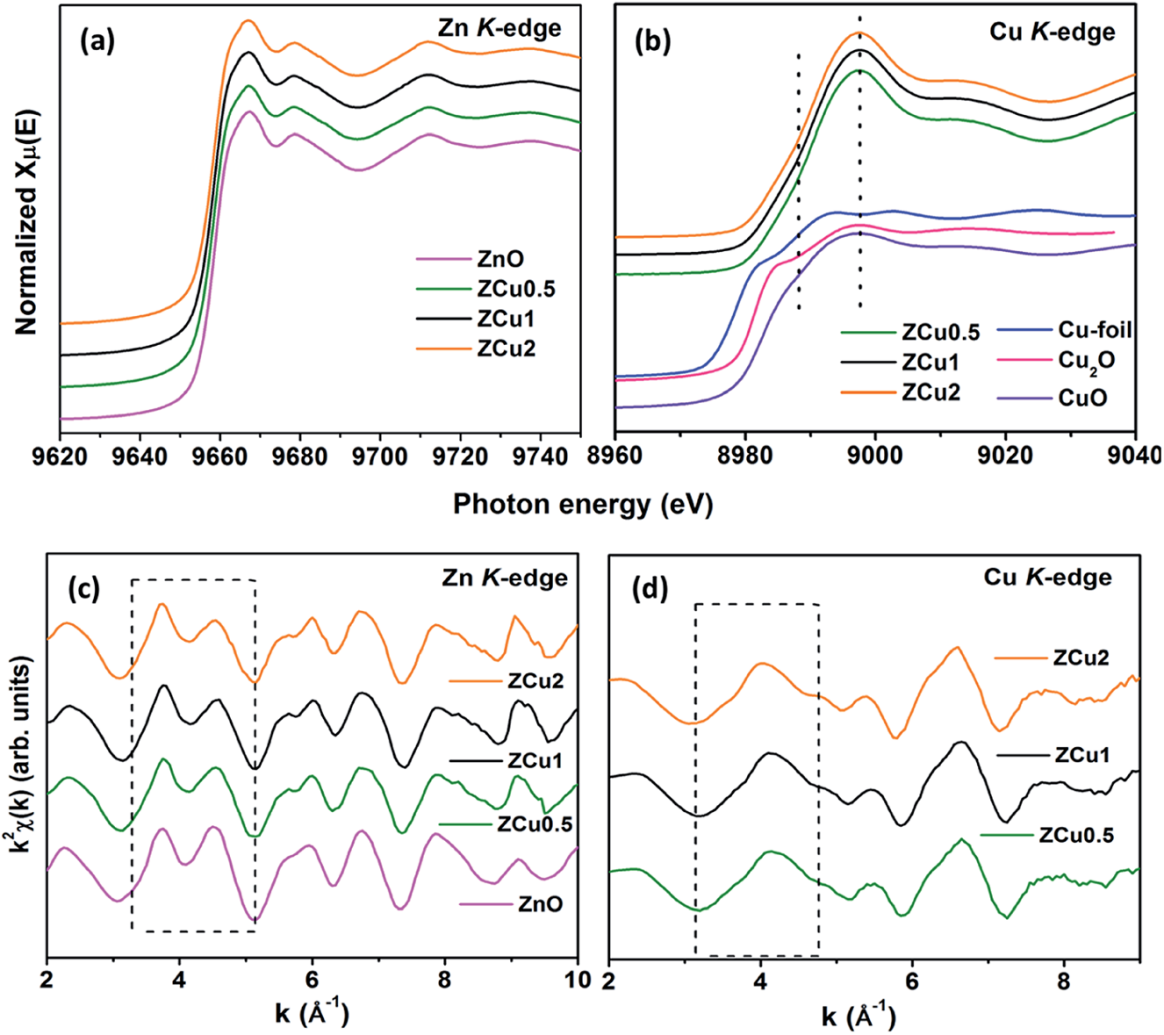
Cu-doped ZnO nanostructure: (a) normalized XANES spectra at the Zn K-edge, (b) normalized XANES spectra at the Cu K-edge, (c) *k*^2^-weighted *χ*(*k*) spectra at the Zn K-edge, and (d) *k*^2^-weighted *χ*(*k*) spectra at the Cu K-edge. The dashed box encloses a feature that is characteristic of the ZnO structure. The data are vertically shifted for better understanding.

To determine the local atomic structure around absorbing atoms, EXAFS measurements at the metal (Cu,Zn) K-edge were performed for various synthesized ZnO samples. XANES spectra of Cu doped ZnO nanostructures measured at Zn and Cu K-edges are shown in Fig. 8(a) and (b), respectively along with the standard Cu metal foil, Cu_2_O and CuO. The XANES at the Zn K-edge for Cu doped samples are found to have a similar trend in spectral features with increasing Cu ion concentration in the ZnO matrix. Similarly, at the Cu K-edge the samples follow the same spectral features and their absorption edge position matches well with the absorption edge of standard CuO, marked with the dotted line, which indicates the presence of Cu in the +2 oxidation state in the Zn_1−*x*_Cu_*x*_O matrix. Above the absorption edge, the spectral features of Cu doped ZnO samples and standard CuO vary differently, which implies that the local environment of Cu in the ZnO matrix is different from that of Cu in CuO. The pre-edge feature at the Cu K-edge is shown by other groups^67^ whereas in our case, for such low Cu doping concentration X-ray beam line resolution has not resolved this feature well. The EXAFS spectra at Zn and Cu K-edges are shown in Fig S3.† Further, to extract the coordination number (*N*), bond distance (*R*) and Debye–Waller factor (*σ*^2^), the energy dependent absorption coefficient *μ*(*E*) was first converted to the EXAFS function *χ*(*E*), which was then converted in O *k*-space into *χ*(*k*). The *χ*(*k*) functions are Fourier transformed (FT) in *R*-space to generate the *χ*(*R*) *versus R* spectra in terms of the real distances from the center of the absorbing atom. Fig. 8(c) shows the *k*^2^-weighted *χ*(*k*) EXAFS spectra of the Cu-doped ZnO nanostructure at Zn K-edges. The dashed box in the *k*^2^*χ*(*k*) EXAFS panel is used to emphasize the enclosed oscillations which are characteristic of the ZnO structure. The dashed rectangular box again in Fig. 8(d) suggests the formation of the ZnO-like structure around the Cu atom.

**Fig. 9:**
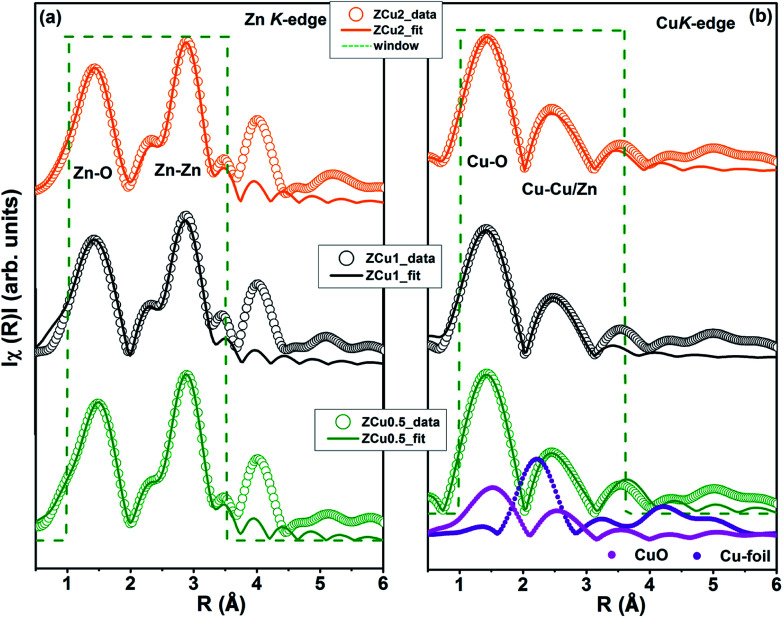
Best fit of radial distribution obtained by FT EXAFS oscillations: (a) at the Zn K-edge (*k* = 3–10 Å^−1^) and (b) at the Cu K-edge in the region *k* = 3–9 Å^−1^.

In the EXAFS simulation performed at the Zn K-edge, the model was assumed as pure ZnO having wurtzite structure with the first prominent peak or first shell in *R*-space corresponding to the Zn–O bond where Zn is coordinated with first 4 O atoms and the second prominent peak corresponds to the Zn–Zn bond where Zn is coordinated with 12 second nearest Zn atoms. The structural lattice parameters used in the simulation are those obtained from Rietveld XRD refinement. The fitting of the Zn K-edge for pure ZnO is shown in our previous work,^68^ also shown in Fig. S2.† These parameters were further used for the fitting at the Zn K-edge for Cu doped samples. Fig. 9(a) shows the (*R*) *vs. R* plot at the Zn K-edge FT in the range *k* = 3.0–10.0 Å^−1^. The fitting was performed in the phase uncorrected *R*-space range 1–3.5 Å while the resultant fitted parameters are given in phase corrected data. The best fit results for *N*, *R* and *σ*^2^ at the Zn K-edge are summarized in Table 1. Fig. 9(b) shows the *χ*(*R*) *vs. R* plot at the Cu K-edge, FT in the range *k* = 3.0–9.0 Å^−1^ along with the standard CuO and Cu metal foil. For Cu K-edge EXAFS simulation, it is assumed that a few Zn atoms in the ZnO have been replaced by Cu atoms in the wurtzite structure. The first shell in *R*-space corresponds to the Cu–O bond whereas the second shell corresponds to the Cu–Cu/Zn bond space. Similar to the Zn K-edge, the S_O_^2^ and Δ*E*_o_ values for the first two shells were extracted from the fitting of CuO which were found to be 5 ± 1 eV and 0.7 ± 0.1, respectively. These values were kept constant for Cu doped ZnO samples. The best fit results for the Cu K-edge are summarized in Table 2. From the *χ*(*R*) *vs. R* plot at the Cu K-edge, it is observed that the Cu–O bond distance for the Cu-doped ZnO series and standard CuO is different which marks the fact that the CuO phase is not present in the prepared samples. Further from the Zn K-edge fitting results, it is observed that the Zn–O bond distance decreases from 1.967 Å for pure ZnO to 1.939 Å for ZCu2 while the Zn–Zn bond distance shows an increase with increasing Cu concentration in the ZnO matrix. The decrease in Zn–O bond distance on Cu doping is expected because of the smaller ionic radii of tetrahedrally coordinated Cu^2+^ (0.071 nm) when compared with Zn^2+^ (0.074 nm). On the other hand, Cu K-edge fitting results show an increase in Cu–O and Cu–Cu/Zn bond distance with increasing Cu concentration in the ZnO matrix. In addition to this, the Debye–Waller factor (*σ*^2^) is found to increase on Cu doping indicating increased local disorder near the Zn absorber atom in the ZnO lattice. Moreover, the decrease in coordination numbers (*N*) of Zn–O, Zn–Zn, Cu–O and Cu–Cu/Zn further implies that Cu doping results in an increase of vacancies in the ZnO matrix, supporting the PL results.

**Table 1:** Different parameters such as *N*, *R* and *σ*^2^ obtained after EXAFS data fitting for the first and second shell at the Zn K-edge for Cu-doped ZnO samples

Path	Parameter	ZnO	ZCu0.5	ZCu1	ZCu2
Zn–O	*N*	4	3.8	3.8	3.6
	*R* (Å)	1.967	1.940	1.949	1.939
	*σ* ^2^ (Å^2^)	0.002	0.004	0.004	0.006
Zn–Zn	*N*	12	11.9	11.8	11.6
	*R* (Å)	3.178	3.197	3.206	3.246
	*σ* ^2^ (Å^2^)	0.003	0.006	0.005	0.008

**Table 2:** Different parameters such as *N*, *R* and *σ*^2^ obtained after EXAFS data fitting for the first and second shell at the Cu K-edge for Cu-doped ZnO samples

Path	Parameter	ZnO	ZCu0.5	ZCu1	ZCu2
Cu–O	*N*	—	3.7	3.3	3.2
	*R* (Å)	—	1.913	1.918	1.945
	*σ* ^2^ (Å^2^)	—	0.002	0.003	0.005
Cu–Cu/Zn	*N*	—	11.3	10.8	10.4
	*R* (Å)	—	2.821	2.951	3.075
	*σ* ^2^ (Å^2^)	—	0.003	0.008	0.01

### 3.5 Magnetic measurements

**Fig. 10:**
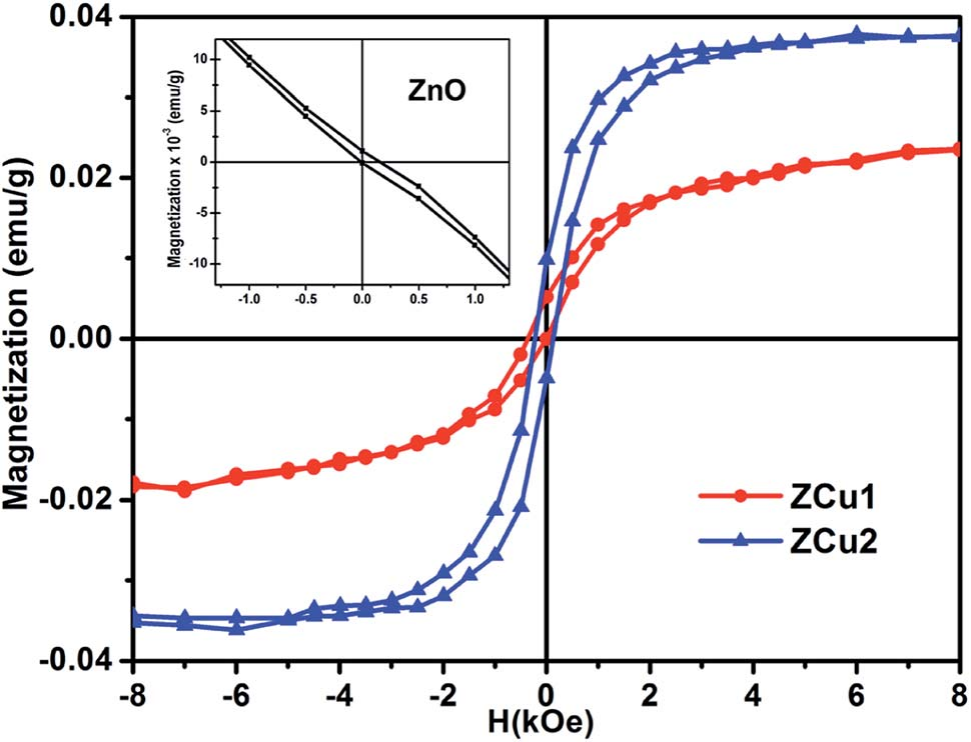
Room temperature hysteresis loop (M–H curve) for the Cu-doped ZnO nanostructure. Inset shows the M–H curve of pure ZnO.^69^

Room temperature magnetic measurements of pure ZnO and Cu doped ZnO through VSM have been carried out. The magnetization as a function of the applied magnetic field (M–H) at 300 K for Cu doped ZnO at different Cu ion concentrations is plotted. The experimental results show that the M–H curve for pure ZnO is a characteristic of diamagnetic materials, as shown in the inset of Fig. 10. The magnetic results of ZnO are also shown in our previous study.^69^ Even at low temperatures of 5 K, pure ZnO shows diamagnetism upon magnetization reversal.^22^ Anupama *et al.* reported the diamagnetic behavior of ZnO nanoparticles at 300 K and 2 K synthesized *via* a chemical method route with a crystallite size of 27 nm.^15^ In a similar manner, Vijayaprasath *et al.* also observed diamagnetism in ZnO nanoparticles of crystal size ∼30 nm prepared by the co-precipitation method.^70^ However, Shoushtari *et al.* studied the effect of ZnO grain size on magnetic properties and reported weak ferromagnetism at a low magnetic field of 0–2000 Oe in ZnO nanoparticles with a grain size of 36 nm.^71^ Based on these results, the origin of ferromagnetism in the pure ZnO nanostructure is still not clear. In Fig. 10, the M–H hysteresis curve is identified at room temperature for Cu doped ZnO samples and it is observed that for low Cu concentration in Zn_1−*x*_Cu_*x*_O (*x* = 0.01 and 0.02), the M–H hysteresis curve shows a weak ferromagnetic signature at 300 K (after the removal of the diamagnetic component). As discussed earlier, for a wide range of applications, a DMS material should possess *T*_C_ above room temperature (300 K) and it is clear from the RT M–H hysteresis curve of Cu : ZnO systems that these synthesized materials through the sol–gel route can retain the FM characteristic at room temperature. The values of fundamental magnetic properties such as coercivity (*H*_c_), retentivity (*M*_r_) and saturation magnetization (*M*_s_) for Cu doped ZnO systems have been noted. It is observed that with increasing Cu content in the ZnO matrix, the value of retentivity changes from 0.005 emu g^−1^ for ZCu1 to 0.010 emu g^−1^ for ZCu2 and the coercivity shows an increase from 170 Oe for ZCu1 to 225 Oe for ZCu2. These magnetic properties (*M*_r_, *H*_c_) of a material depends upon various factors which include the crystallite size, particle shape, stoichiometry, and dopant type. The saturation magnetization, *M*_s_, was also found to increase with an increasing dopant concentration in Zn_1−*x*_Cu_*x*_O. The saturation magnetization observed in our case for ZCu2 is ∼0.04 emu g^−1^. Hammad *et al.* noticed a RT *M*_s_ value of 0.69 emu g^−1^ for 1% Cu-doped ZnO nanoparticles^72^ and Herng *et al.* recorded a *M*_s_ of 0.03 *μ*_B_/Cu atom in ZnO : Cu films.^73^ The variation in saturation magnetization and magnetic moment values depends upon a number of factors. The dopant ion type, synthesis environment, crystal anisotropies, and crystallinity are responsible for the experimentally observed magnetic properties.^74^ In addition to this, micro-structural parameters like creation of strain, vacancies or defect states also affects the magnetic behavior. Further, the magnetic moment per Cu atom is calculated using eqn (5)5
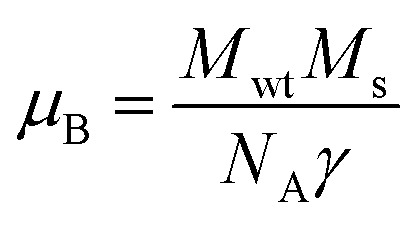
where *μ*_B_ is the Bohr magnetron, *M*_wt_ is the molecular weight of the compound, *M*_s_ is the saturation magnetization and *γ* is the conversion factor and is equal to 9.27 × 10^21^ erg per Oe. The values of *M*_s_ and magnetic moments for ZCu1 and ZCu2 are listed in Table 3. It is observed that the value of *μ*_B_ decreases with increasing dopant ion concentration from 1% to 2% in the ZnO matrix. Also, the calculated magnetic moment in Cu-doped ZnO is less than the theoretically calculated values for Cu with tetrahedral geometry *i.e.*, Cu^2+^ (1.8 *μ*_B_). Theoretical simulations for the 3d transition metal doped ZnO system showed that the magnetic properties are strongly affected by the relative location between TM atoms^75^ and moreover, the nano-structured nature of the ZnO and the weaker inter-particle exchange interactions could be the reason for the low magnetic moment in our case.

**Table 3:** *M*_s_ values and magnetic moment per Cu atom for the Cu-doped ZnO nanostructure

Sample name	*M*_s_ (emu g^−1^)	*μ*_B_/dopant atom
ZCu1	0.023	0.034
ZCu2	0.039	0.029

The origin of RTFM in the Cu doped ZnO nanostructured system is still under a controversial debate. There are a number of reasons proposed in the literature for the ferromagnetic properties in these transition metal doped ZnO systems. First, at the nanoscale level the formation of Cu clusters or oxides results in secondary phase formation and due to the high surface to volume ratio, the surface effects are pronounced for the ZnO nanostructure. Second, defect states or vacancies or magnetic impurities are present in the synthesized samples. The first point has not been established in Cu : ZnO systems up to 2% of doping as the presence of Cu impurities in respective ZnO systems has been ruled out by the XRD and NEXAFS analysis. Moreover, CuO is also antiferromagnetic in nature with a Neel temperature of 230 K.^76^ Therefore, the RTFM observed in the Cu-doped ZnO system is intrinsic instead of impurity induced. Considering the second point, the presence of vacancies or intrinsic defects such as Zn_i_, *V*_O_, O_i_, and *V*_Zn_ plays a crucial role in the FM interaction for TM doped ZnO based DMS systems^77,78^ and the visible emission in PL measurements showed the existence of intrinsic defect states in Cu : ZnO systems. Supporting the PL results, the NEXAFS study at Cu and O K-edges revealed hybridization of Cu (3d) states with the O (2p) state, which indicates the fact that oxygen vacancies are responsible for the observed RTFM. Long-range interactions are required for achieving high-temperature FM ordering in TM doped ZnO based DMS systems and these interactions are mediated by defect-induced states.^79^ A number of theories have been proposed that include the (i) mean-field Zener model, (ii) Ruderman–Kittel–Kasuya–Yosida (RKKY) carrier-mediated interactions, (iii) direct interactions such as double and super-exchange, and (iv) donor impurity band exchange model in which there is an indirect exchange mediated through shallow donor electrons forming bound magnetic polarons (BMPs) and resulting in FM ordering in DMSs. Among the various theories and ideas, RKKY interactions include free conduction band electrons and ZnO being a semiconductor cannot change into metal at such low doping concentration.^45^ Thus, the RKKY model does not fit in the synthesized Cu : ZnO system. Moreover, the doping concentration in our case is only up to 2% and the free carriers offered by the doping element are too limited to form a permanent FM coupling based on the carrier-mediated interactions, while the presence of oxygen vacancies is confirmed by NEXAFS measurements due to the substitution of Zn^2+^ by Cu^2+^ ions in their respective systems. Hence the intrinsic ferromagnetism observed is most likely mediated by oxygen vacancies rather than carriers. The most discussed defect induced ferromagnetic mechanism in DMSs is the BMP model. As discussed earlier, Coey *et al.*'s spin–split impurity band model explains ferromagnetic coupling mediated by polaronic percolation of BMPs produced by defects such as oxygen vacancies.^26^ BMPs consist of bound electrons or holes along with the spins of the TM ions within a hydrogenic Bohr orbit of radius *r*_H_ = 0.76 nm for ZnO. The sp–d coupling between bound polarons and dopant ions (Cu) results in a significant alignment of spins giving rise to the net ferromagnetism. Along with the oxygen vacancies, researchers also reported zinc vacancies contributing to the intrinsic exchange interactions in TM doped ZnO *via* BMP formation.^80^ Zhu *et al.* assigned the observed RTFM to oxygen vacancy induced Cu^2+^ ferromagnetic coupling in Cu-doped ZnO nanoparticles synthesized through the hydrothermal method.^81^ In addition to this, it is also noticed that with increasing Cu doping concentration, the *M*_s_ value increases but the *μ*_B_ value shows a decrease. It is more likely for the doped Cu^2+^ ions to occupy the nearest neighbor positions with increasing concentration. The exchange interaction between the Cu–Cu pair is antiferromagnetic which results in a decreased magnetic moment per doped atom. Besides this, with the increased dopant concentration in the ZnO matrix, there are chances of overlapping of hydrogenic Bohr orbitals with the randomly positioned defects resulting in an overall reduced magnetic moment.^82,83^ Further, effort has been made to fit the M–H curve to the BMP model represented by eqn (6)^84^

**Fig. 11:**
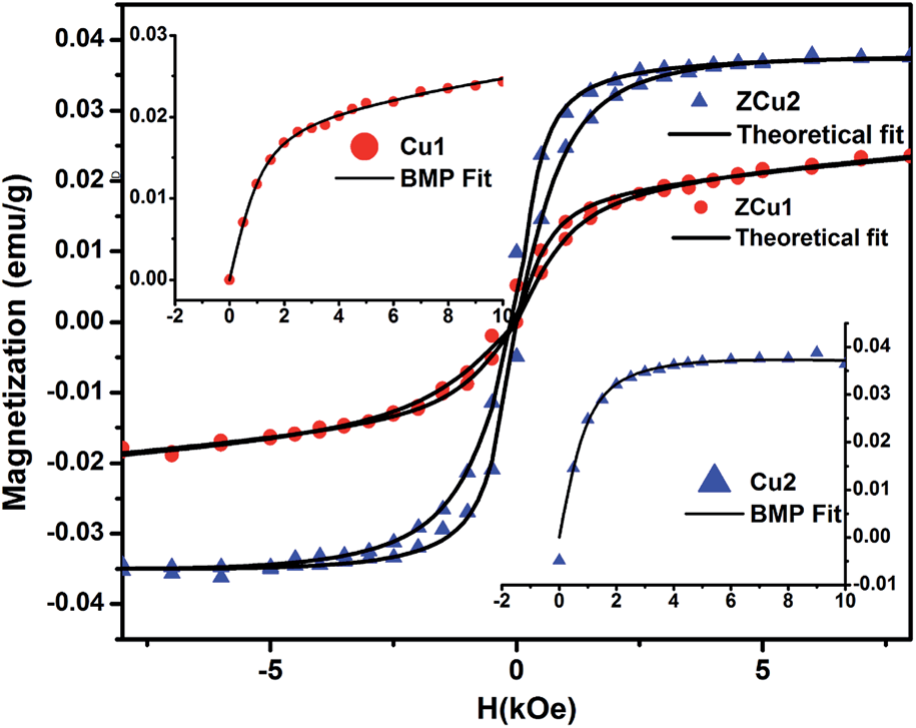
Experimental M–H curve (solid patterns) along with fitted curves (solid black line) for ZCu1 and ZCu2. Inset shows the BMP model fitting.

6 *M* = *M*_o_*L*(*x*) + *χ*_m_*H*

where the first term is acquired from the BMP contribution and the second term corresponds to the paramagnetic contribution. Here *M*_o_ = *Nm*_s_, *N* is attributed to the number of BMPs and *m*_s_ is the effective spontaneous magnetic moment per BMP. The Langevin function *L*(*x*) = coth(*x*) − 1/*x* with *x* = *m*_eff_*H*/*k*_B_*T* where *m*_eff_ is the true spontaneous moment per BMP. At high temperatures *m*_eff_ is approximately equal to *m*_s_. The M–H curve for ZCu1 and ZCu2 fitted with the BMP model is shown in Fig. 11 and it is noticed that the experimental data closely follow the fitted data, which further shows that the BMP model is highly acceptable to explain the observed RTFM in Cu : ZnO systems. From the fitting, the BMP concentration parameter *N* is found to be in the order of 10^16^ cm^−3^, which is required for long-range ferromagnetic ordering and is also in agreement with the reported papers.^85^

Room temperature magnetic properties of TM-doped ZnO have been studied widely and in Table 4 a few recent findings are compiled which also convey the fact that observed RTFM is due to defect mediated exchange interactions. Fe-doped ZnO^91^ and (Fe,Ni) co-doped ZnO^92^ also exhibted FM ordering mediated through oxygen vacancies. Ali *et al.* showed experimental and theoretical results emphasizing RTFM in Cu-doped ZnO due to the exchange interaction between Cu^2+^–Cu^2+^ ions mediated by zinc vacancies.^93^

**Table 4:** Some recently reported TM-doped ZnO as a DMS material

Compound	TM composition (*x*)	Synthesis method	Observations
(Zn,Co)O^15^	5%, 10%	Chemical method	FM is due to the exchange interaction between Co (d) electrons and free carriers generated due to Co-doping
(Zn,Cu)O^81^	1%	Hydrothermal route	FM is attributed to oxygen vacancies
(Zn,Co)O^86^	0 < *x* < 0.1	Solid-state reaction	Antiferromagnetic coupling between Co ions at higher magnetic fields
(Zn,Ni)O^87^	0.05–0.2	Microwave assisted combustion	Weak FM at 300 K, FM due to oxygen and/or Zn vacancies
(Zn,Mn)O^88^	1 : 5, 4 : 5	Wet chemical and post hydrogen annealing	Annealed samples show FM, Mn–H–Mn bridge structure and the Mn–Mn exchange interaction
(Zn,Mn)O^89^	0.02–0.08	Solid-state reaction	Intrinsic FM due *V*_O_ and/or defects
(Zn,Cr)O^90^	1–5%	Sol–gel dip-coating	FM is due to bound magnetic polaron (BMP) interactions mediated through oxygen vacancies
(Zn,Fe)O^91^	0–20 at%	Sol–gel spin coating	FM is defect mediated by exchange interaction of oxygen vacancies and Fe ions
(Zn,Cu)O^93^	0.05–10%	Magnetron sputtering	Overlapping of BMPs causes the alignment of their spins, resulting in long-range ferromagnetic order
(Fe,Ni) co-doped^92^	1% Fe, 2% Ni	Hydrothermal method	FM due to oxygen vacancies associated with the BMP model
ZnO			

## 4 Conclusions

In this paper, we experimentally revealed the existence of RTFM in Cu-doped ZnO nanostructures and used the BMP model to understand the cause of ferromagnetism. Rietveld refined XRD confirms the formation of the hexagonal wurtzite phase in Cu : ZnO systems up to a certain doping concentration. The secondary oxide phase of Cu appeared in the ZnO matrix at 3% ad 5% of Cu concentration in the present synthesis route and conditions. Approximately the same crystallite size is observed even after Cu doping. From the FE-SEM micrographs, the growth mechanism is put forward giving relevant facts about the formation of bullet like nanostructures in ZnO and Cu doped ZnO systems. UV-DRS measurements showed band gap decreases on Cu substitution, whereas, from PL studies an appreciable increase in the visible emission is observed with the increase in Cu concentration, which further shows the increase in intrinsic defect states/vacancies in the synthesized Cu-doped ZnO samples. Further, NEXAFS measurements at the O K-edge revealed the hybridization of Cu doped 3d states with the O (2p) state, which indicates the fact that oxygen vacancies are responsible for the observed RTFM. The Cu L_3,2_-edge also showed no signs of the presence of Cu metallic clusters (measurement taken up to 2%) and also confirmed the presence of Cu in the +2 oxidation state. Local electronic structure investigations performed through EXAFS data fitting revealed the variation in Zn–Zn, Zn–O, Cu–O, and Cu–Cu/Zn bond distances on Cu-doping. A systematic increase in disorder (*σ*^2^) and decrease in coordination number (*N*) near the Zn sites are also observed on Cu doping. The study explains that intrinsic exchange interactions arising from oxygen vacancy assisted BMPs are responsible for the RTFM in Zn_1−*x*_Cu_*x*_O (*x* < 2%) and proves the Cu : ZnO system to be a DMS having potential applications in the fields of optoelectronics and spintronics.

## Conflicts of interest

There are no conflicts to declare.

## Supplementary Material

NA-002-D0NA00499E-s001
