# Supplementary material for: Influence of Cu doping on the local electronic and magnetic properties of ZnO nanostructures
Source: Nanoscale Adv. 2020 Aug 28;2(10):4450–63. doi: 10.1039/d0na00499e (PMC9417303; doi:10.1039/d0na00499e)
Supplement: NA-002-D0NA00499E-s001 [file NA-002-D0NA00499E-s001.pdf]

Electronic Supplementary Material (ESI) for Nanoscale Advances.
This journal is © The Royal Society of Chemistry 2020

Influence of Cu doping on local electronic and magnetic properties of ZnO nanostructures

Richa Bhardwaj[a*], Amardeep Bharti[b*], Jitendra Pal Singh[c], Keun Hwa Chae[c] and Navdeep Goyal[a]

[a] *Department of Physics, Panjab University, Chandigarh, 160-014, India. Tel: +91 7589297611; E-mail: rbhardwaj.phy@gmail.com*

[b] *Material Science Division, Inter-University Accelerator Center, New Delhi 110067, India. E-mail: abharti_phy@yahoo.com*

[c] *Advanced Analysis Center, Korea Institute of Science and Technology, Seoul, 02792, Republic of Korea*

Table S1 represents the average crystallite size, lattice parameter and various reliability (R) factors obtained after Rietveld refinement of Cu doped ZnO samples.

Table S1: Structure parameters and R-factors obtained after refinement.

Sample name	Avg. Crystallite size (nm)	Lattice Parameters		R-factors			Atomic position				
		a (Å)	*c* (Å)	R_p	R_e	χ^2	Atoms	Wyckoff Position	x	y	z
ZnO	36 ±1	3.2492(0.0001)	5.2063(0.0002)	12.4	4.1	1.1	Zn<br>O	2(b)<br>2(b)	0.3333<br>0.3333	0.6667<br>0.6667	0<br>0.3798
ZCu0.5	43 ±1	3.2507(0.0001)	5.2074(0.0002)	13.0	3.82	1.8	Zn/Cu<br>O	2(b)<br>2(b)	0.3333<br>0.3333	0.6667<br>0.6667	0<br>0.3799
ZCu1	41 ±1	3.2504(0.0001)	5.2059(0.0002)	6.8	3.63	1.5	Zn/Cu<br>O	2(b)<br>2(b)	0.3333<br>0.3333	0.6667<br>0.6667	0<br>0.3819
ZCu2	40±1	3.2546(0.0001)	5.2137(0.0003)	11.9	3.61	2.1	Zn/Cu<br>O	2(b)<br>2(b)	0.3333<br>0.3333	0.6667<br>0.6667	0<br>0.3823

To confirm the element stoichiometry, the ZCu0.5 (0.5% Cu doping) sample is investigated through Energy-dispersive X-ray spectroscopy (EDS). Elemental analysis of ZCu0.5 confirmed the presence of Cu dopants and their composition in ZnO system, shown in Fig.S1.

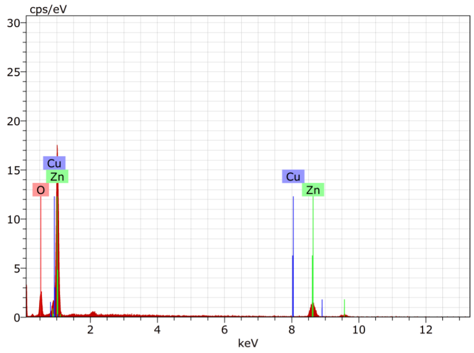

Element	Series	unn. C [wt.%]	norm. C [wt.%]	Atom. C [at.%]	Error (3 Sigma) [wt.%]
Oxygen	K-series	14.38	13.68	39.30	7.81
Zinc	K-series	90.54	86.12	60.55	10.47
Copper	K-series	0.21	0.20	0.15	0.25
	Total:	105.13	100.00	100.00	

Fig. S1: EDS spectrum and table of composition for ZCu0.5.

Fig.S2 (a) shows the normalized EXAFS and the XANES spectrum at Zn *K*-edge for pure ZnO sample. The spectral feature at the absorption edge at ~ 9670 eV is attributed to the electron transition from Zn(1*s*) to Zn(4*p*) states. Further, in order to take care of the oscillations in the absorption spectra the energy dependent absorption coefficient μ(E) is first converted to EXAFS function χ(E) expressed through Eq. 1[1]

$$\boldsymbol{\chi}(\mathbf{E}) = \frac{\boldsymbol{\mu}(\mathbf{E}) - \boldsymbol{\mu}\mathbf{o}(\mathbf{E})}{\boldsymbol{\Delta\mu}\mathbf{o}(\mathbf{E})} \qquad (\mathbf{1})$$

where μo(E) is the absorption seen in absence of EXAFS effects, Δμo(E) is the measured jump in μ(E) at threshold energy Eo and division by Δμo(E) normalizes the EXAFS oscillations per atom. The χ (E) is converted to the photoelectron wave number dependent absorption function χ(k) using the given below Eq. 2

$$k = \sqrt{\frac{2m(E - Eo)}{h^2}} = 0.263\sqrt{E - Eo} \qquad (2)$$

here m is the electron mass, E and Eo are in eV and k is inverse angstroms, $Å^{-1}$. At last, χ(k) is weighted by k^2 to amplify the oscillation at high k and the $\chi(k)k^2$ functions are Fourier transformed (FT) in R-space to generate the χ(R) versus R spectra in terms of the real distances from the center of the absorbing atom. The EXAFS simulation can be performed using Athena, Artemis software available in IFEFFIT packages[2,3]. For quantitative analysis, to extract the various parameters such as coordination number (N), bond distance (R) and disorder (Debye-Waller) factor (σ^2), the function χ(k) can be expressed as a sum over all interference patterns arising from neighboring atoms.

$$\chi(k) = \Sigma \frac{N_j}{kR_j^2} S_o f_j(k) e^{-2k^2\sigma_j^2} e^{\frac{-2R_j}{\lambda(k)}} sin[2kR_j + \delta_j(k)] \qquad (3)$$

Eq. 3 describes the EXAFS signal with structural parameters, which are of great interest for depicting coordination geometry information[4]. Paths with similar kind of scattering atoms and a comparable path length have been arranged under the index *j*. $f_j(k)$ signifies the energy dependence of the photoelectron scattering and λ(k) denotes the energy-dependent mean free path of the photoelectron. The structural parameter N_j is equivalent to the number of backscattered atoms or coordination number. $\delta_j(k)$ is the phase change in the electron wave due to the central and neighboring atom potentials. The first exponential term $e^{-2k2\sigma j2}$ is called as the Debye-Waller factor, where σ^2_j is the variation analogous to absorbing and scattering atom distance distribution (R_j) due to thermal vibrations or structural disorder. The Debye factor represents the fact that each absorber-scatterer distance will contribute to the EXAFS oscillations. The second exponential term $e^{-2R j /\lambda (k)}$ indicates the loss of photoelectrons due to inelastic scattering, where *λ(k)* is the energy-dependent electron mean free path. So the EXAFS

contribution from every backscattered atom is a damped sine wave in k-space having amplitude and phase both dependent on wave vector k. In addition to the above, So^2 signifies the constant amplitude reduction factor which includes the inelastic loss processes.

Fig.S3 (b) shows the Fourier Transform (FT) of EXAFS oscillations performed using FEFF9.05 Artemis simulation tool, also reported previously [5].

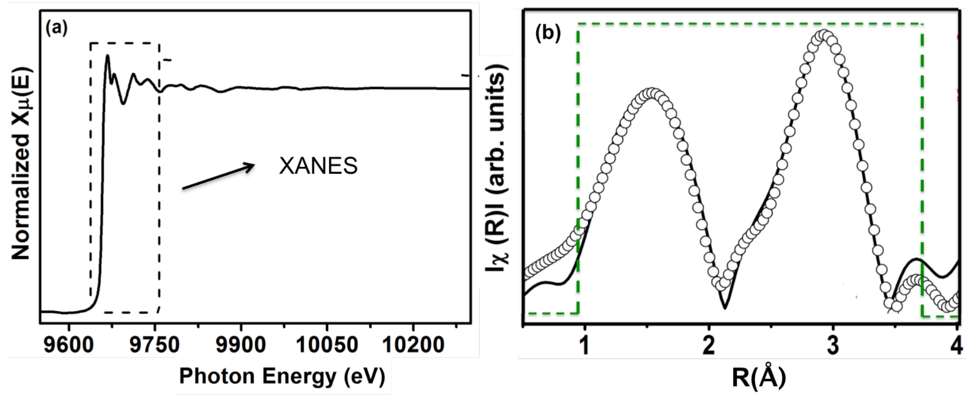

Fig. S2: (a) Normalized Xμ(E) spectrum at Zn K-edge of pure ZnO. Dotted box represents the XANES part. (b) Radial distribution obtained by Fourier Transform of EXAFS oscillations with S_O^2 and absorption edge energy shift (E_o) values 0.72 and 4.78 eV, respectively [5].

Fig.S3 (a) and (b) shows the normalized EXAFS spectra Zn and Cu K-edges for Cu doped ZnO nanostructure at different Cu concentration. The CuO and Cu foil are used as reference samples to observe the behavior of oscillations above absorption for Cu K-edge.

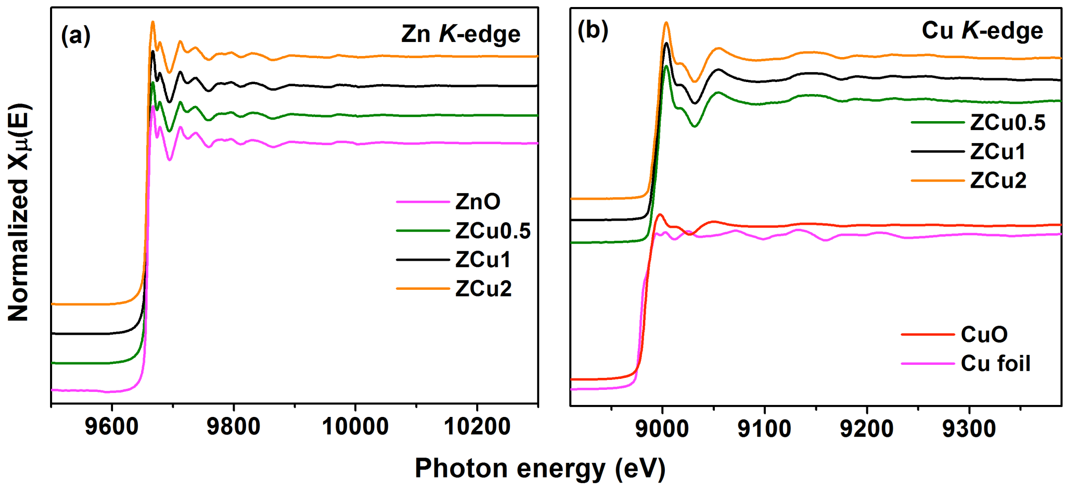

Fig. S3: EXAFS spectra measured for Cu-doped ZnO nanostructure. (a) Normalized Xμ(E) spectra at Zn K-edge, (b) Normalized Xμ(E) spectra at Cu K-edge.

References:

1. D. C. Konigsberger and R. Prince, X-Ray Absorption:Principles, Applications, Techniques of EXAFS, SEXAFS and XANES, Wiley, New York, 1988.
2. J. J. Rehr, J. J. Kas, F. D. Vila, M. P. Prange and K. Jorissen, Parameter-free calculations of X-ray spectra with FEFF9, *Phys. Chem. Chem. Phys.*, 2010, **12**, 5503-5513.
3. J. J. Rehr, Theory and calculations of X-ray spectra: XAS, XES, XRS, and NRIXS, *Radiat. Phys. Chem.*, 2006, **75**, 1547-1558.
4. M. Newville, Fundamentals of XAFS, *Rev. Mineral Geochem.*, 2014, **78**, 33-74.
5. R. Bhardwaj, K. H. Chae and N. Goyal, Electronic structural study of defect-induced magnetism in Co doped ZnO nanostructure, *Vacuum*, 2020, **178C** 109446.
